# Evidence for a Global Sampling Process in Extraction of Summary Statistics of Item Sizes in a Set

**DOI:** 10.3389/fpsyg.2016.00711

**Published:** 2016-05-13

**Authors:** Midori Tokita, Sachiyo Ueda, Akira Ishiguchi

**Affiliations:** ^1^Faculty of Health Sciences, Mejiro UniversitySaitama, Japan; ^2^Faculty of Core Research, Ochanomizu UniversityTokyo, Japan

**Keywords:** summary statistical representation, ideal observer analysis, statistical efficiency, size averaging, variance discrimination

## Abstract

Several studies have shown that our visual system may construct a “summary statistical representation” over groups of visual objects. Although there is a general understanding that human observers can accurately represent sets of a variety of features, many questions on how summary statistics, such as an average, are computed remain unanswered. This study investigated sampling properties of visual information used by human observers to extract two types of summary statistics of item sets, average and variance. We presented three models of ideal observers to extract the summary statistics: a global sampling model without sampling noise, global sampling model with sampling noise, and limited sampling model. We compared the performance of an ideal observer of each model with that of human observers using statistical efficiency analysis. Results suggest that summary statistics of items in a set may be computed without representing individual items, which makes it possible to discard the limited sampling account. Moreover, the extraction of summary statistics may not necessarily require the representation of individual objects with focused attention when the sets of items are larger than 4.

## Introduction

When we encounter a group of people, we can figure out the average size, the average age, and/or the average emotional expression of the group, or even variations among these. Such instant estimations might help determine our next course of action in the situation.

Many studies have shown that people accurately perceive and estimate the statistical properties of a set of items or events, even claiming the existence of summary statistical representations (Chong and Treisman, 2003; Alvarez, 2011). Observers are able to quickly and accurately extract average values over a range of visual properties, including size (Ariely, 2001, 2008; Chong and Treisman, 2003, 2005; Oriet and Brand, 2013), brightness (Bauer, 2009), orientation (Dakin and Watt, 1997; Parkes et al., 2001), and emotional expression (Haberman and Whitney, 2009, 2011). Moreover, this ability is not limited to static and simultaneous events; it is also observed in dynamic objects such as expanding and contracting circles (Albrecht and Scholl, 2010). Recent studies have shown that the perceptual system's ability to represent statistical properties is not limited to visual properties but also comprises auditory mechanisms such as extracting frequency information and estimating variance from sound sequences (Piazza et al., 2013; Byrne et al., 2014).

Although there is a general understanding that human observers can accurately represent sets of features, many questions on how summary statistics are computed remain unanswered. Three possibilities on the computations of statistics have been proposed: (1) summary representations are computed without computing individual items, (2) representations of individual items are computed first and then combined to form a summary representation, and (3) only a few items in a set are sampled and included in the extraction of summary statistics.

The first and second proposals predict there to be specialized summary statistical representation mechanisms that are separate from the mechanisms mediated to represent individual objects. Conforming to this argument, many studies have proved that when attention is distributed across a set of similar items, people can extract the average size of all items without relying on focused attention to individual items in the set (Chong and Treisman, 2003, 2005; Alvarez and Oliva, 2008; Corbett and Oriet, 2011; Im and Halberda, 2013). The third proposal claims that it is possible to accurately estimate the average size by sampling a few items in a set using focused attention. Some modeling research has shown that a sampling strategy reasonably predicts the approximate levels of performance exhibited by observers in studies of average-sized objects of perception (Myczek and Simons, 2008; Marchant et al., 2013).

Neither the proponents of the summary representation mechanisms nor those of the limited sampling strategy have excluded or refuted the opposing argument. Rather, they demonstrate the need for further investigation on the processes of human performance in summarizing statistical mechanisms (Ariely, 2008; Simons and Myczek, 2008). Recent studies have attempted to examine the nature of such summary representations using various experimental methods (Solomon et al., 2011; Jacoby et al., 2013; Attarha et al., 2014; Huang, 2015). For example Solomon et al. suggested that some observers might use at least three items in estimates of average size using the simulation method. In short, it is necessary to further examine if people estimate summary statistics by using global information concerning all items in a set, or information concerning only a limited number of items in a set.

The present study investigated sampling properties of visual information used by human observers in deriving summary statistics of items in a set using an ideal observer analysis. We tested two types of summary statistics: average size and size variance of visual item sets. In Experiment 1, we estimated the size discrimination threshold of human observers in each experimental condition to determine values for free parameters used in simulating the performance of the ideal observer models. In Experiment 2, we measured performance on a size-averaging task for each ideal observer model and for human observers. Then, we compared the performance of the ideal observer of each model to the performance of human observers to evaluate which model could accurately predict how human observers derive the average size of items in a set. In Experiment 3, we measured performance on a variance discrimination task for each ideal observer model and for human observers. Then, we compared the performance of the ideal observer of each model to the performance of human observers to evaluate which model could adequately predict how human observers derive the size variance of items in a set.

While comparing, we used a statistical efficiency analysis that allows direct comparison of efficiencies among different models that represent different uses of information. Statistical efficiency is a relative index for the sampling rate of information in a given task. Many studies have utilized efficiency to investigate how the visual system uses available information and reveals the characteristics of human performance (Burgess and Barlow, 1983; Watamaniuk, 1993; Ikeda and Ishiguchi, 2004; Tanaka and Ishiguchi, 2006). Based on the three proposals mentioned above, we presented three corresponding models of ideal observers to perform the task: a global sampling model without sampling noise (GSM1), global sampling model with sampling noise (GSM2), and limited sampling model (LSM). First, we explained the ideal observer models in more detail, and then demonstrated how we calculated the statistical efficiency.

### Ideal observer models

**Figure 1:**
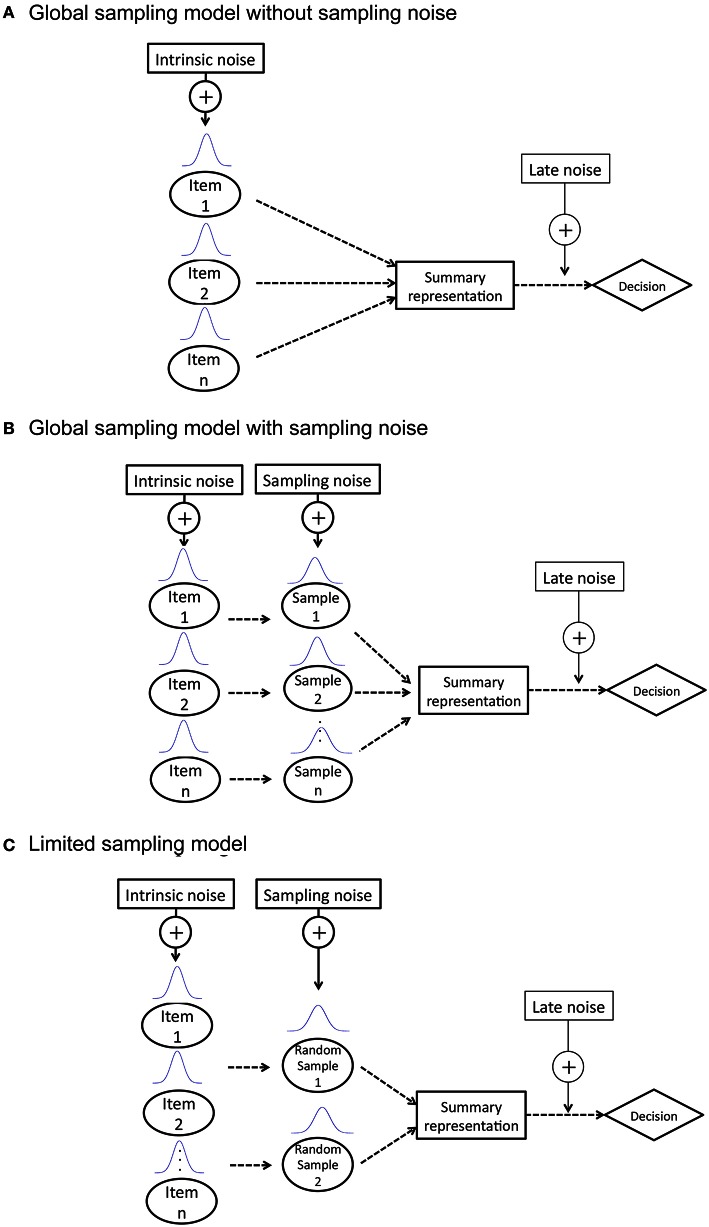
**Description of three ideal observer models: (A)** global sampling model without sampling noise (GSM1); **(B)** global sampling model with sampling noise (GSM2); **(C)** limited sampling model (LSM).

An ideal observer is a theoretical device that performs a given task in an optimal manner with the available information and some specified constraints. The theory of ideal observers has proven to be a powerful and useful tool in vision research, and has been applied to a wide range of studies (Geisler, 2003, 2011; Knill and Saunders, 2003; Yakushijin, 2007). We presented three ideal observer models, as mentioned above, to perform two types of summary statistics (i.e., size average and size variance): GSM1, GSM2, and LSM. Figure 1 shows diagrams of each model. Each model comprises three processes in common: a sampling process, a summary representation process, and a decision process. There are three types of noises involved in a given process: an intrinsic noise added to each item in a set prior to the individual sampling (σ_Intrinsic_), a sampling noise added to each sampled item in a set prior to summary representation (σ_Sample_) and a late noise added to an estimated average value (σ_Late_) such as memory noise. Since the late noise is assumed to be added equally in each model, we do not discuss it further in the description of the three models.

GSM1 posits that summary representation is extracted without computing individual items and is used for calculating summary statistics. Thus, there is only intrinsic noise added to each item in a set.

GSM2 posits that representations of individual items are extracted first and then combined to form a summary representation. The sample noise is added to each individual item before the formation of the summary representation. The value of the noise depends on the set size, since it has been predicted that the noise accompanied with the representation of each individual item increases as the number of items increases, since each item receives less attention (Palmer, 1990; Franconeri et al., 2007).

LSM posits that observers sample a few items randomly chosen from a set to represent their summary statistics.

The LSM ideal observer randomly sampled four items from a set, except for two items sampled in the set size two condition. This is because it has been widely accepted that three to four items may be simultaneously processed, involving preattentive mechanisms of limited capacity (Mandler and Shebo, 1982; Cowan, 2000). In addition, Myczek and Simons (2008) showed that observers could accurately estimate the average of the set by sampling two items and estimating the average of those two items alone.

The performance of each model, discriminability $d_{Ideal}^{\prime }$, was derived using the Monte Carlo method. Free parameters of the models, standard deviation (SD) for intrinsic noise for each item (σ_Intrinsic_) and SD for sampling noise of each set size (σ_SampleN_) were fitted separately for each experiment.

### Calculation of statistical efficiency

We calculated statistical efficiencies using the following method. First, we prepared two stimuli: a set of multiple items and a single test item. Both stimuli consisted of solid circles with a given diameter. The diameter of the test item was larger or smaller than the average size of items in a set. We added independent lognormal noise ln*N*(ln*D*, $\sigma_{c}^{2}$) to the diameter of individual items in the set. No noise was added to the test item. The observer's task was to determine which item (the average size of items in a set or the test item) was larger in size, using the two alternative forced choice (2AFC) procedure. Ideal observers of each model utilized the values of information available in each model. The discriminability for each ideal observer ($d_{Ideal}^{\prime }$) was obtained by performing the Monte Carlo simulation. The discriminability for human observers ($d_{H}^{\prime }$) was determined by performing the same 2AFC task and calculated as follows:

(1) $${d^{\prime }}_{H}=\sqrt{2}z_{\text{c}},$$

where z_*c*_ is the z-value transformed from the observer's percentage of correct estimations. Statistical efficiency *F* was defined by the square of the ratio of these two scores as

(2) $$F={\left({d\prime }_{H}/{d\prime }_{Ideal}\right)}^{2}.$$

The details of the calculation of statistical efficiency have been discussed in Barlow (1978) and Watamaniuk (1993).

It has been shown that human observers make perceptual decisions with efficiency of up to ~50% (Barlow, 1978; Barlow and Reeves, 1979; van Meeteren and Barlow, 1981; Tanaka and Ishiguchi, 2006). If statistical efficiency is larger than 100%, we could infer that the model may not describe the appropriate process with which to perform the task. The fluctuation of efficiency has also been useful to explore the characteristics of the human sampling strategy (Cormack et al., 1994; Tanaka and Ishiguchi, 2006). In this study, we consider the characteristics of statistical efficiency in evaluating each model.

## Experiment 1: size discrimination experiment

The purpose of this experiment was to obtain the value of free parameters σ_*Intrinsic*_ and σ_SampleN_. To obtain the parameter σ_*Intrinsic*_, we measured size discrimination threshold in a single item condition. To obtain the parameter σ_SampleN_, we measured the discrimination threshold for one item in a set in each of four set size conditions (2, 4, 9, and 16) using a post-cue procedure.

Intrinsic noise resides within a sensory system and limits the detectability and discriminability of signals. The standard deviation of the discriminability of signals is expected to represent the volume of intrinsic noise by co. To measure the intrinsic noise, we conducted the size discrimination experiment of a single item (i.e., Task 1).

Intrinsic noise resides within a sensory system, thus it will be attached to an item representation irrespective of presentation conditions. In contrast, sampling noise will be added to each item in a set when the visual system (observer) holds the representation of each item until a following task, causing uncertainty in the sampling process. Note that observers should distribute their attention to the whole array of items, rather than one particular item in a set. In order to estimate the volume of sampling noise in each set size, it is necessary to obtain the discrimination threshold of one (called a target) of the items in the set, while observers distribute their attention to the whole array. With this purpose, observers should be informed of the target item immediately after the offset of the array. To realize the condition experimentally, we introduced a post position-cue method in which a post-cue appeared on the position of a target item immediately after the offset of an array (Task 2). The standard deviation of the discriminability of the target item was expected to represent the volume of sampling noise. We predicted that the discriminability of the target item would decrease with set size, so as in the visual task (Mazyar et al., 2012).

### Method

#### Participants

There were four observers, author MT and three experienced psychophysical observers, TT, YA, and SU. All had normal or corrected-to-normal vision. Informed consent was obtained form all observers. This experiment was performed in accordance with the Code of Ethics of the World Medical Association (Declaration of Helsinki).

#### Design

**Figure 2:**
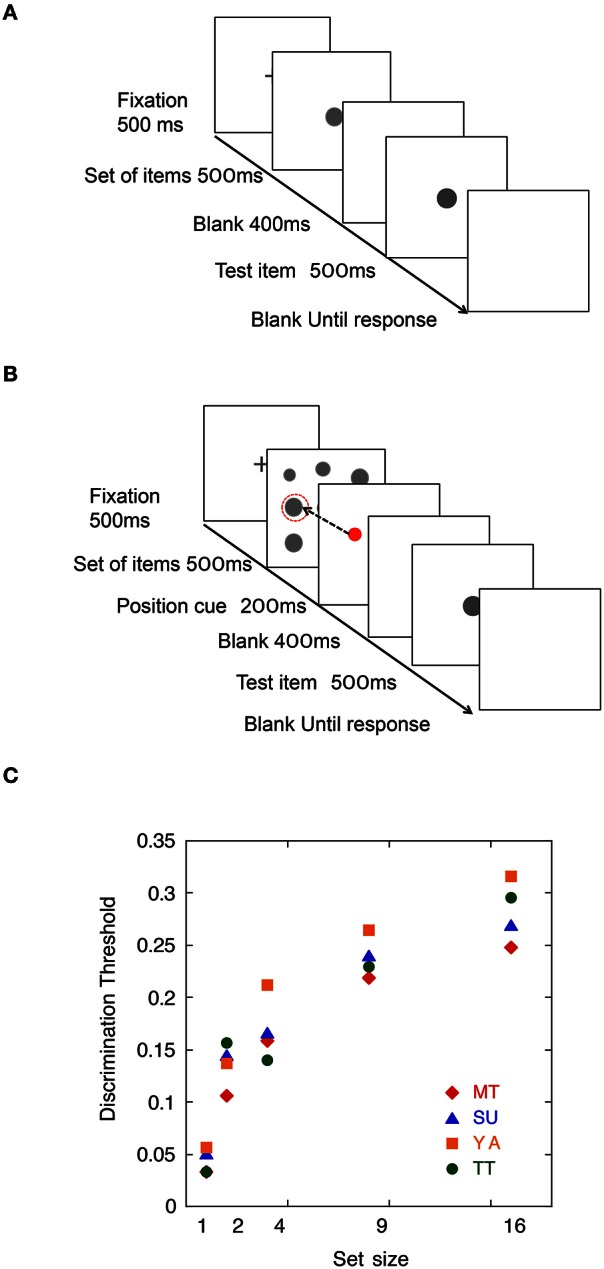
**(A)** Schematic view of a trial sequence in Task 1. **(B)** Schematic view of a trial sequence in Task 2. **(C)** Means of size discrimination threshold as a function of set size in Experiment 1.

To obtain the parameter σ_Intrinsic_, we measured size discrimination thresholds of a single item (i.e., Task 1). Figure 2A shows a schematic view of the trial sequence. To obtain the parameter σ_SampleN_, we tested a size discrimination threshold of an item randomly selected in an item set of four set sizes (i.e., Task 2). There were four set size conditions: 2, 4, 9, and 16. Figure 2B shows a schematic view of the trial sequence.

#### Stimuli

The items consisted of light gray dots on a dark gray background. In Task 2, the items were arranged on the array of an m × m cell matrix: 2 × 2 (set size = 2, 4), 3 × 3 (set size = 9), and 4 × 4 (set size = 16). Each item was displayed at the center of each cell with a position jitter. The size of the display area varied between ~3.4 and 4.2° of visual angle. The average diameters of dots varied between ~0.47 and 0.57° of visual angle. In each trial, all the dots shown were randomly scaled by a small multiplicative factor to discourage participants from basing their judgments on previously seen stimuli. Three multiplicative factors (1, 1.1, 1.2) were used and the same factor scaled all items in any one trial.

A lognormal Gaussian noise ln*N*(lnD, $\sigma_{\text{c}}^{2}$) was added to the diameter of each item of a set independently. It has been established that a lognormal distribution of circle diameters will produce a Gaussian distribution of discriminable sizes after logarithmic transduction (Solomon et al., 2011). Thus, in an attempt to create normal distributions of transduced size, we use the lognormal distributions of circle diameter. In this experiment, σ_c_ was set to 0.2.

#### Procedure

In Task 1, the test item and a target item were presented in one interval of the two interval trial. Each trial started with a fixation cross for 500 ms. Then, the item (i.e., test or target item) was presented first for 500 ms, the other item (i.e., test or target item) was presented for 500 ms after the ISI of 400 ms. The observers' task was to decide which item was larger in size.

In Task 2, a set of items was presented in the first interval and a test item was presented in the second interval. One of the items was a target item, which needed to be compared with the test item. The set of items, consisting of a given number of dots, was presented in the first temporal interval of the two interval trial. The test item, consisting of one dot, was presented in the second interval. Each trial started with a fixation cross for 500 ms. Then, the items in the set were presented first for 500 ms. A red dot position cue, indicating the target, was presented for 200 ms after the item set. The test item was presented for 500 ms after the ISI of 400 ms. The observers' task was to decide which item, the target item in the set or the test item, was larger in size. In both experiments, the QUEST procedure (Watson and Pelli, 1983) adaptively determined the JND at which the observer judgment was 84% correct. There were 40 trials in a block. The observers performed 4 to 5 blocks for each set size condition. There were 10 practice trials for each set size. Discrimination threshold was calculated by dividing the JND by the size of the target item.

A 2AFC procedure was used. When observers thought that the target item in the set was larger than the test item, they pressed “1.” When they thought that the test item was larger than the target item, they pressed “3.” No feedback about the correctness of responses was provided.

#### Apparatus

The stimuli were presented on the screen of a Mitsubishi 17 inch monitor. The monitor was driven by a Mac Pro computer that also performed all timing functions and controlled the course of the experiment. Display resolution was 1024 × 758 pixels. Participants viewed the screen with both eyes and were seated ~115 cm from the monitor, fixed in position with a chin rest.

#### Results

Results are shown in Figure 2C. The discrimination threshold for a single item condition for observers MT, SU, YA, and TT were 0.033, 0.051, 0.056, and 0.033, respectively. These values were used as the parameters for intrinsic noise (σ_Intrinsic_). In Task 2, the discrimination thresholds of observers MT, SU, YA, and TT for the target in set sizes 2, 4, 9, and 16 were 0.106, 0.159, 0.219, and 0.248; 0.144, 0.167, 0.240, and 0.270; 0.137, 0.212, 0.264, and 0.316; and 0.157, 0.140, 0.229, and 0.296, respectively. These values were used for the parameters for item noise (σ_SampleN_) for each set size.

#### Discussion

The discrimination thresholds for single items were 0.033–0.056. They were mostly consistent with the results of previous studies (Morgan, 2005; Nachmias, 2011), which tested the size discrimination threshold of circles. These values were used for the intrinsic noise (σ_Intrinsic_) for each observer.

The results of Task 2 showed that the discrimination threshold increased as the number of items increased as anticipated. The results were consistent with the prediction that the value of noise depended on set size since each item received less attention (Palmer, 1990; Franconeri et al., 2007). Each value was used as the sampling noise (σ_SampleN_) for each set size for each observer in following simulations.

## Experiment 2: size averaging experiment

In Experiment 2, we investigated sampling properties of visual information used by human observers in deriving the average size of items in a set. First, we tested human observers' ability to discriminate between the estimated average size of a set and a test item. Next, we simulated the performance of the size-averaging task for each ideal observer model using the parameters obtained in Experiment 1. Then, we compared the performance of the ideal observer of each model to the performance of human observers to evaluate which model could predict human behavior.

### Method

#### Participants

The same observers participated as in Experiment 1.

#### Stimuli

Stimuli were the same as in the Size Discrimination Experiment except that no position cues were presented in the interval between the item set and the following test item.

#### Design

There were two independent variables in the experiment. The first variable was the number of items in a set; there were four set sizes: 2, 4, 9, and 16. The second variable was the level of difference between average size of a set and test item. There were two size difference levels: ± 0.08 (hard) and ± 0.12 (easy) relative to the average size. Concretely, when the difference level was ± 0.08, the area size of the test item was 8% larger or smaller than the expected average size of the set item in a given trial.

A set of items was presented in the first interval and a test item was presented in the second interval. Each condition had 200 trials, resulting in 1600 trials in total. Total trials consisted of 10 blocks. Each block had 160 trials [10 repetitions × 4 set size × 2 levels × 2 directions of test size (smaller or larger)]. Participants performed five blocks in each experimental session, and conducted two sessions in total. The set size and the level of difference were blocked and the order of trials were randomly mixed. Observers were given 20 practice trials before the actual experiment began.

#### Procedure

**Figure 3:**
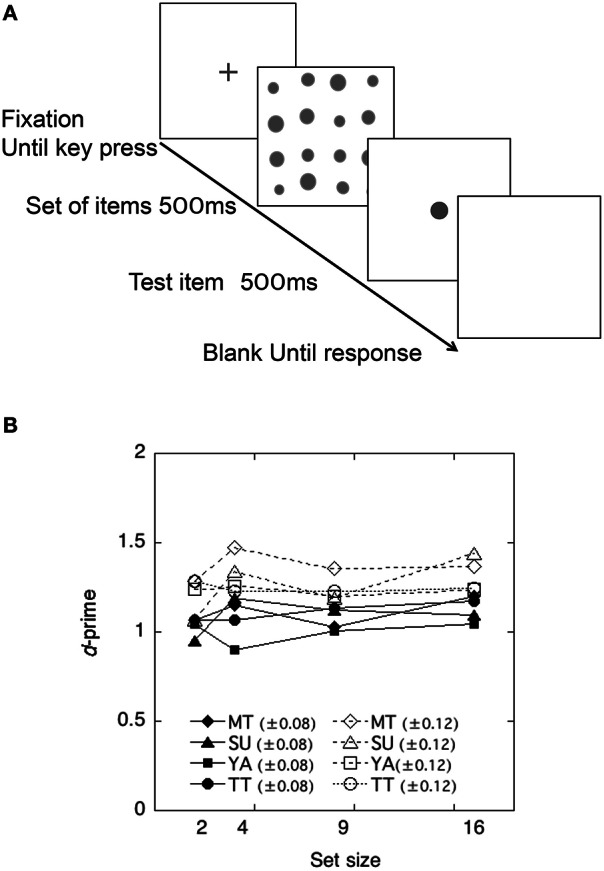
**(A)** Schematic view of a trial sequence in the Size Averaging Experiment. **(B)** Discriminability of each observer as a function of the set size in the size-averaging task in Experiment 2.

A schematic view of the stimulus presentation is shown in Figure 3A. Each trial started with a fixation cross for 500 ms. The items in a set were presented first for 500 ms and the test item for 500 ms after an intermission of 400 ms. The observers' task was to decide whether the test item was larger or smaller than the average size of items in a set.

A 2AFC (larger or smaller) was used. When observers thought that the test item was smaller than the average size of items in a set, they pressed “1.” When they thought that the test item was larger than the mean size of a target set, they pressed “3.” No feedback about the correctness of responses was provided.

#### Results

The performance of each observer is shown in Figure 3B. Each discriminability $d_{H}^{\prime }$ was calculated using the Equation (1) and plotted as a function of the set size. As shown in Figure 3B, discriminability appeared to be unaffected by the number of items in a set, being consistent with findings in previous studies (Ariely, 2001; Chong and Treisman, 2003; Tokita and Ishiguchi, 2014).

To test whether and how the averaging performance differed across difference levels and across set size, a 2 difference level × 4 set size repeated measures ANOVA was conducted on the individual *d'*. This yielded a significant main effect of difference levels, *F*_(1, 31)_ = 23.80, *p* < 0.01. The effects of set size and interactions were not significant, *F*_(3, 31)_ = 2.43, *p* > 0.05 and *F*_(3, 31)_ = 0.35, *p* > 0.05. Thus, the results demonstrated that observers showed higher discriminability (±0.12) when the difference between the average size of items and the test item was larger.

### Statistical efficiencies and evaluation of models

**Figure 4:**
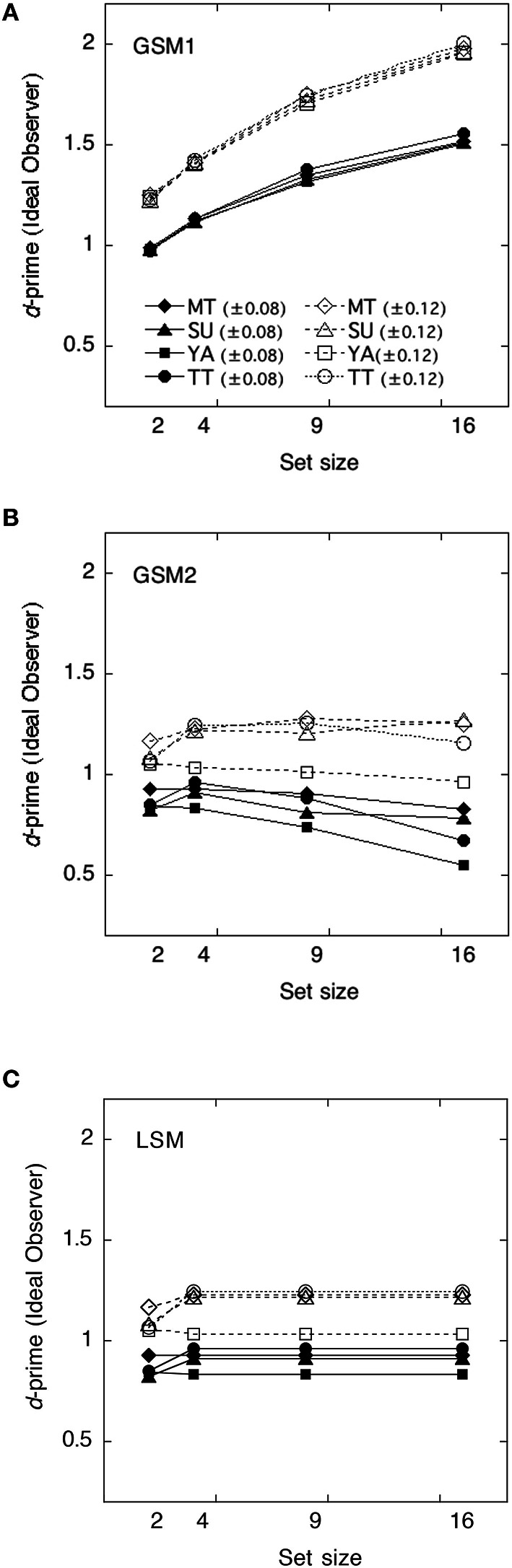
**The ideal observer's discriminabilities for each model as a function of set size. (A)** Global sampling model without sampling noise (GSM1); **(B)** Global sampling model with sampling noise (GSM2); **(C)** Limited sampling model (LSM).

**Figure 5:**
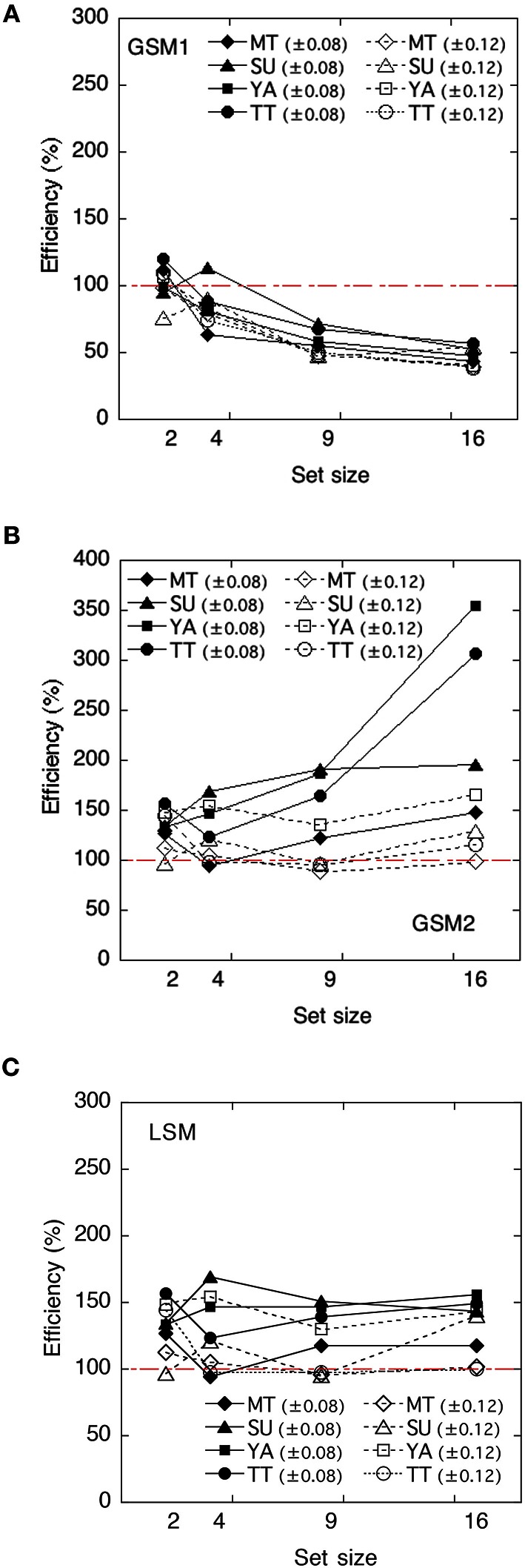
**The human efficiencies of each model as a function of set size. (A)** Global sampling model without sampling noise (GSM1); **(B)** Global sampling model with sampling noise (GSM2); **(C)** Limited sampling model (LSM). The efficiency scores are reported as a percentage.

We calculated the ideal observers' discriminabilities $d_{I}^{\prime }$ for each model by using the parameters obtained in Experiment 1. Discriminabilities for each model are shown in Figure 4. Derivation of each model is shown in Appendix in Supplementary Material. As the value of sampling noise increases with set size, $d_{I}^{\prime }$ in GMS2 declines with set size. We calculated statistical efficiencies for each observer using Equation (2). These are presented in Figure 5 as a function of set size. To test whether the statistical efficiency was above or below 100%, we conducted a one-sample *t*-test to compare the statistical efficiencies of each condition with 100% criterion.

The efficiencies of the GSM1 were not significantly different from 100% at a set size of 2, *t*_(7)_ = 0.42, *p* > 0.1, whereas they were significantly below 100% at a set size of 4, *t*_(7)_ = −3.09, *p* < 0.05; 9, *t*_(7)_ = −44.34, *p* < 0.01; and 16, *t*_(7)_ = −20.69, *p* < 0.01, respectively. Thus, the efficiencies varied across set sizes. When the set sizes were 2, the efficiencies approximated 100%, whereas when they were 9 and 16, the efficiencies approximated 60%.

The efficiencies of the GSM2 were significantly above 100% across set size: at the set size of 2, *t*_(7)_ = 4.59, *p* < 0.01; at 4, *t*_(7)_ = 2.70, *p* < 0.05; at 9, *t*_(7)_ = 2.41, *p* < 0.05; and at 16, *t*_(7)_ = 2.70, *p* < 0.05.

The efficiencies of the LSM were also significantly above 100% across set size: at the set size of 2, *t*_(7)_ = 4.59; *p* < 0.01; at 4, *t*_(7)_ = 2.70, *p* < 0.05; at 9, *t*_(7)_ = 2.62, *p* < 0.05; and at 16, *t*_(7)_ = 4.06, *p* < 0.01.

## Discussion

As the statistical efficiencies of GSM2 and LSM exceeded 100% and the performance of human observers was higher than those of the models, we could assume that human observers did not adopt these strategies. On the other hand, the efficiencies of the GSM1 approximated 100% when the set size was 2 and approximated 60% when it was 9 and 16. When the set size was four, efficiency marked the value between 60 and 100%. Thus, we can assume that GSM1 may be an appropriate model for the human observers in deriving the average of item sets. This suggests that the summary statistics representation might derive the average size of items in a set without representing the size of individual items.

These results were consistent with the claim that observers can estimate with high accuracy the average size of a set of items, even when they seem unable to report the size of individual items in the set (Ariely, 2001). In other words, the results disagree with the claim that the average of the set could be accurately estimated by sampling as few as one or two items, and estimating the average of those items (Simons and Myczek, 2008). Observers are not strategically subsampling when they compute the mean size, especially in the case where the number of items is large, such as 9 and 16. There might be individual differences in the way observers access averages.

## Experiment 3: variance discrimination experiment

In Experiment 3, we investigated sampling properties of visual information used by human observers in deriving size variance of items in a set. First, we tested the performance of the variance discrimination task for human observers. Next, we simulated the performance of the variance discrimination for each ideal observer model using the parameters obtained in Experiment 1. Then, we compared the performance of the ideal observer of each model to the performance of human observers to evaluate which model could predict human behavior.

### Method

#### Participants

The same observers participated as in the previous experiments.

#### Stimuli

**Figure 6:**
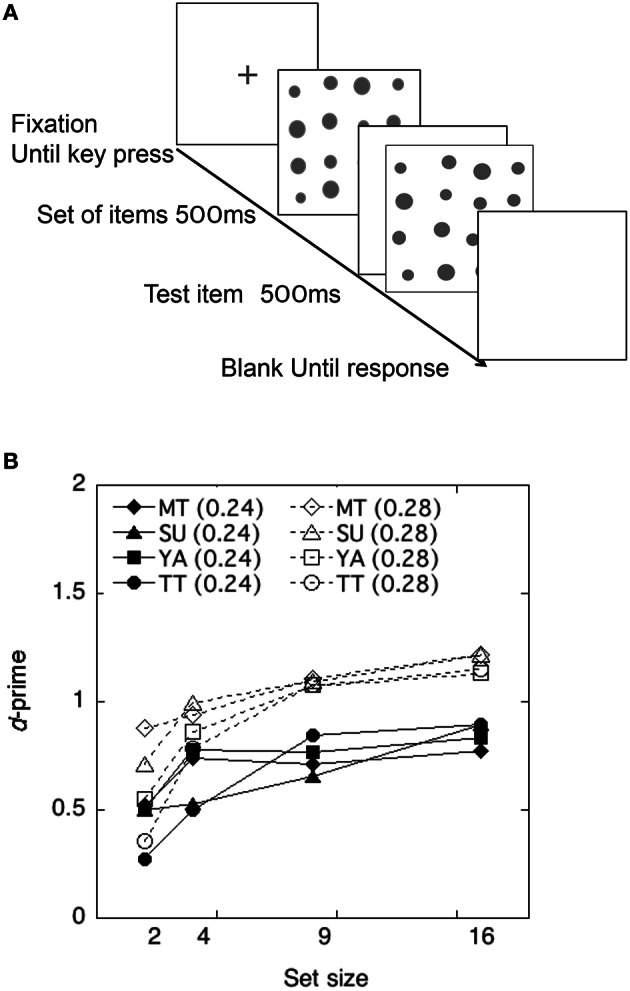
**(A)** Schematic view of a trial sequence in the variance estimation experiment. **(B)** Discriminability of each observer as a function of set size in the variance discrimination task in Experiment 3.

A schematic view of the stimulus presentation is shown in Figure 6A. Stimuli were the same as in previous experiments except that both display (i.e., standard and comparison set) comprised item sets.

#### Design

There were two independent variables. These were varied within observers. The first variable was the number of items in a set; there were set sizes of 2, 4, 9, and 16, the same as in Experiment 2. The second variable was the level of difference between generated variance of a comparison set and a standard set. We added independent lognormal noise ln*N*(ln*D*, σ^2^) to the diameter of individual items in the comparison and standard sets. The mean standard deviasion of a standard set was 0.2. There were two levels of expected standard deviation in a comparison set: 0.24 (hard) and 0.28 (easy). The sequence of trials was completely randomized within a block; the standard stimuli came first in half of the trials, and second in the remaining trials.

Each condition had 200 trials, resulting in 1600 trials in total. Total trials comprised 10 blocks. Each block had 160 trials [10 repetitions × 4 set size × 2 levels × 2 temporal orders (first or second)]. The participants performed five blocks in each experimental session, and two sessions in total. The set size and the level of difference were blocked and the order of trials was randomly mixed. Observers were given 20 practice trials before the actual experiment began.

#### Procedure

Each trial started with a fixation cross for 500 ms. The first items in the set were presented first for 500 ms and the second for 500 ms after an intermission of 400 ms. The observers' task was to decide which set, first and second, had larger variance. A 2AFC procedure (first or second) was used. When observers thought that the first set had larger variance than the second set, they pressed “1.” When they thought that the second set had larger variance than that of the first set, they pressed “3.” No feedback about the correctness of responses was provided.

#### Results

The performance of each observer is shown in Figure 6B. The discriminabilities $d_{H}^{\prime }$*s* were calculated using the Equation (1) and plotted as a function of the set size. As shown in Figure 6B, discriminability appeared to be slightly increased with the number of items in a set, especially when the difference in variance was large.

To test whether and how the performance of variance discrimination differed across difference level and across set size, repeated measures ANOVA with respect to the 2 difference level × 4 set sizes was conducted on the individual *d*′. This yielded a significant main effect of difference level, *F*_(1, 31)_ = 31.81, *p* < 0.01. The effects of set sizes were also significant, *F*_(1, 31)_ = 30.97, *p* < 0.01. The interactions were not significant, *F*_(3, 31)_ = 1.11, *p* > 0.05. A Bonferroni post hoc analysis revealed that in the small difference condition, *d'* at a set size of 2 was significantly smaller than that of 9 (*p* < 0.05) and 16 (*p* < 0.01); in the large difference condition, *d'* at a set size of 2 was significantly smaller than that of 4 (*p* < 0.05), 9 (*p* < 0.01), and 16 (*p* < 0.01), and *d'* at set size of 4 was significantly smaller than that of 16 (*p* < 0.05). All observers showed higher discriminability when the difference in size variance between two sets was larger.

### Statistical efficiencies and evaluation of models

**Figure 7:**
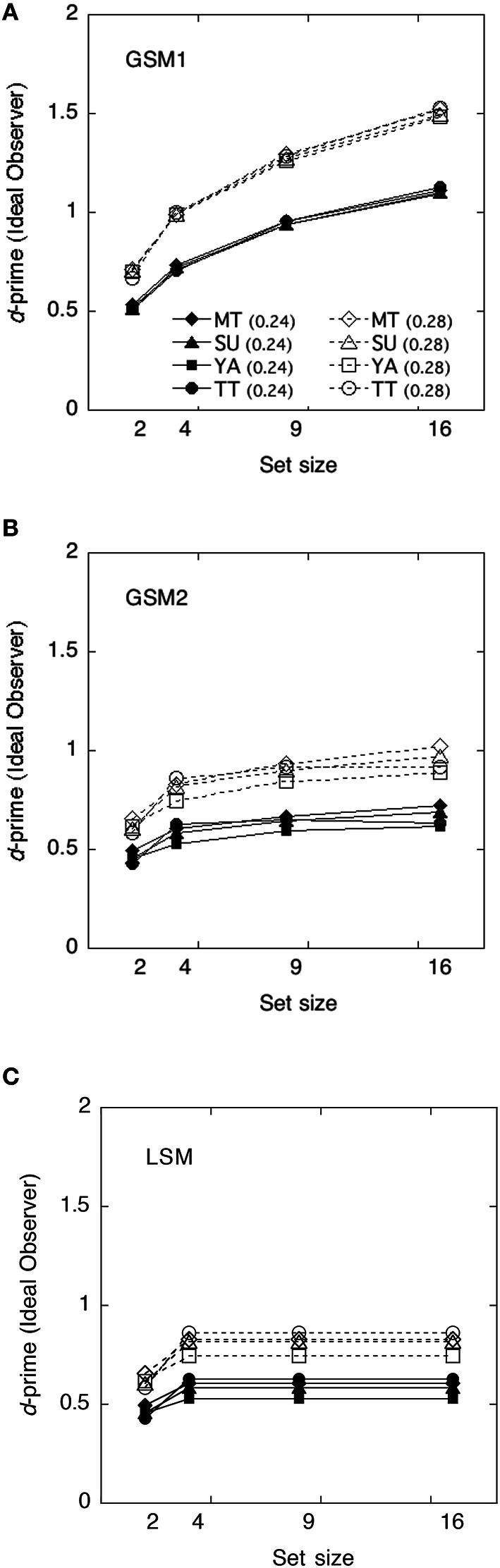
**The ideal observer's discriminabilities of each model as a function of set size. (A)** Global sampling model without sampling noise (GSM1); **(B)** Global sampling model with sampling noise (GSM2); **(C)** Limited sampling model (LSM).

**Figure 8:**
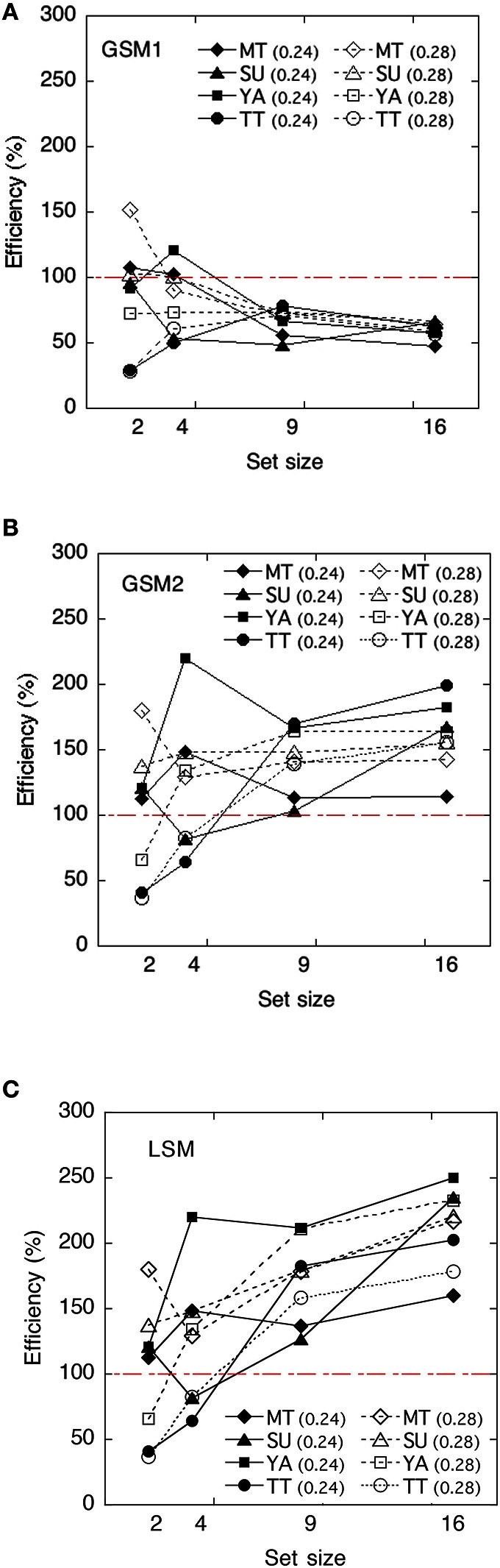
**Human efficiencies of each model as a function of set size. (A)** Global sampling model without sampling noise (GSM1); **(B)** Global sampling model with sampling noise (GSM2); **(C)** Limited sampling model (LSM). Efficiency scores are reported as a percentage.

The ideal observer's discriminabilities for each model were shown in Figure 7. We calculated statistical efficiencies for each observer using the Equation (2). Derivation of each model is shown in Appendix in Supplementary Material. They are presented in Figure 8 as a function of the set size.

To test whether the statistical efficiency was above or below 100%, we conducted a one-sample *t*-test to compare the statistical efficiencies of each condition with that of the 100% criterion.

The efficiencies of the GSM1 were not significantly different from 100% at the set size of 2, *t*_(7)_ = −1.04, *p* > 0.1, and 4, *t*_(7)_ = –2.01, *p* > 0.05, whereas they were significantly below 100% at the set sizes of 9, *t*_(7)_ = −8.97, *p* < 0.01, and 16, *t*_(7)_ = −18.80, *p* < 0.01, respectively. When the set sizes were 2 and 4, the efficiencies approximated 100%, whereas when they were 9 and 16, the efficiencies approximated 60–50%.

The efficiencies of the GSM2 were not significantly different from 100% at the set size of 2, *t*_(7)_ = 0.92, *p* > 0.1, and 4, *t*_(7)_ = 1.47, *p* > 0.1, whereas they were significantly above 100% at the set sizes of 9, *t*_(7)_ = 4.94, *p* < 0.01, and 16, *t*_(7)_ = 6.64, *p* < 0.01, respectively.

The efficiencies of the LSM were not significantly different from 100% at the set sizes of 2, *t*_(7)_ = 0.92, *p* > 0.1 and 4, *t*_(7)_ = 1.47, *p* > 0.1, whereas they were significantly above 100% at the set sizes of 9, *t*_(7)_ = 6.64, *p* < 0.01, and 16, *t*_(7)_ = 10.50, *p* < 0.01, respectively. This means that the human observers did not adopt this strategy.

#### Discussion

As the statistical efficiencies of GSM2 and LSM exceeded 100% and the performance of human observers was higher than that observed in the models, we can assume that human observers did not adopt these strategies. On the other hand, the efficiencies of GSM1 marked 100% and smaller when the set sizes were 2 and 4 except in three cases, and approximated 60–50% when set sizes were 9 and 16. Thus, we can assume that GSM1 may be the appropriate model for human observers in deriving the average of item sets. This suggests that summary statistical representation might derive the variance of items in a set without representing the size of individual items when the set size is 9 or greater. The results were mostly consistent with the results of the averaging task. This suggests that, in the estimation of the size variance of the set, observers are not strategically subsampling. Note that there were large individual differences in efficiencies when the set sizes were 2 and 4. We suspect that it might be difficult for observers to extract the variance when the set sizes are small.

## General discussion

This study investigated sampling properties of visual information used by human observers in deriving summary statistics of items in a set. We introduced three ideal observer models: GSM1, GSM2, and LSM. Two types of summary statistics were measured: average size and size variance of items in a set. In comparing the performance of ideal observers and human observers, we used a statistical efficiency analysis that allowed direct comparison of efficiencies among different models representing different uses of information. In Experiment 1, we obtained two free parameters (i.e., intrinsic noise, σ_Intrinsic_, and sampling noise, σ_SampleN_) that were used for the simulation of each observer. In Experiment 2, we measured the performance of the size-averaging task for each ideal observer model and for each human observer. Then, we compared the performances of the ideal observers with human observers to evaluate which model could predict human behavior well. As the statistical efficiencies of GSM2 and LSM exceeded 100% in most cases, we could infer that human observers did not adopt these strategies. On the other hand, the efficiencies of GSM1 approximated 100% when the set size was 2 and fell below 100% when set sizes were 4 or larger. Thus, we can predict that GSM1 may be the appropriate model for human observers performing the averaging task, indicating that the average values of items in a set may be derived without representing the individual item size. In Experiment 3, we measured the performance of the size variance discrimination task for each ideal observer model and for each human observer. Then, we evaluated each model in the same way as in Experiment 2. As the statistical efficiencies of GSM2 and LSM exceeded 100% in most cases, we can infer that human observers did not adopt these strategies. On the other hand, the efficiencies of GSM1 approximated 100% at a set size of 2, whereas they were below 100% at set sizes of 4, 9, and 16. Thus, we predict that GSM1 might be the appropriate model for human observers in deriving the size variance of a set.

Based on the results of Experiments 2 and 3, we predict that summary statistics may be derived without representing the size of individual items, particularly when set sizes are larger than 4. These results are consistent with the claim that observers can estimate with high accuracy the average size of a set of items, even when they seem unable to report the size of individual items in the set (Ariely, 2001; Chong and Treisman, 2005; Im and Halberda, 2013). In other words, the results disagree with the claim that the average of the set might be accurately estimated by sampling as few as one or two items, and estimating the average of those items. Observers are not strategically subsampling when they compute the mean size, especially in cases where the number of items is large, such as 9 or 16. In their simulation study, Simons and Myczek (2008) demonstrated that sampling one or two individual items from a display to estimate the average of the entire display can result in levels of performance similar to that of human observers. It may be predicted that a discrepancy between Simons and Myczek's study and the present study may be due to considerations of noise: internal noise, memory and decision noise were not taken into account in their studies. On the other hand, Im and Halberda (2013) estimated both the internal noise and the number of samples that affected the performance of size-averaging task and demonstrated that observers sample many more than one or two items from an array. They suggested that extraction of average size relies on a mechanism that is distinct from segmenting individual items, implying a similarity to texture processing.

It should be noted that the present results demonstrate that the size variance of sets may be extracted in a similar way as average size. Our findings suggest that the ability of observers to extract summary statistics is not limited to the average, but may be generalized to size variance estimation.

However, there is an important caveat: the process of summary statistics suggested here may be limited to the area size of solid circles, as far as this study is concerned. Many studies have investigated statistical summary representations for low-level visual features such as spatial frequency, luminance, orientation and motion, and suggested that human observers have the ability to extract summary statistics of these features by pooling across receptors specific to individual elements (Simons and Myczek, 2008). As Simons and Myczek (2008) suggested, area size is not analogous to motion or orientation but is instead thought to be an intrinsic property of an object, because there are no specific receptors for individual absolute size. Since testing whether the process of summary statistics of area size generalizes to other low-level visual features is outside the scope of the present study, we will need to test this possibility in the future.

Next, we discuss similarities and differences between the statistical efficiencies in the size-averaging task and those in the variance discrimination task in more detail. The efficiencies of set sizes 9 and 16 in those tasks were quite similar in two ways. First, they were stable across observers and stimuli levels. Second, the efficiencies value ranged from 50 to 60%. The results suggest that the same amount of visual information was used in the different tasks. This implies that a common mechanism may be involved in the two different tasks. This mechanism may be what some researchers call “summary statistical representation.” At the same time, there is a substantial difference in efficiencies at the set sizes of 2 and 4 between the size-averaging task and the variance task. The efficiencies at the set sizes of 2 and 4 in the averaging task were larger than those at the set sizes of 9 and 16, and the tendency was relatively stable across observers and stimuli levels, whereas those in the variance task demonstrated large individual variations, particularly at the set size of 2. Some studies have suggested that changes in efficiency may be the result of changes in human sampling strategies (Barlow, 1978; van Meeteren and Barlow, 1981; Tanaka and Ishiguchi, 2006). In line with this argument, we predict that the strategy of the observers at set sizes of 2 and 4 differs from that of the large number of items and that strategies differed between observers in the variance task.

Why were the efficiencies at set sizes 2 and 4 larger than those at set sizes 9 and 16? One might suspect that memory load for a small number of items might be lower than that for a large number of items, as observers can process the size of each item attentively in the former case. The lower memory load in the smaller set size would yield the smaller memory noise, resulting in smaller degradation in available information. Thus, the efficiencies in the smaller set size were marked larger than they were in the larger set size condition. However, if this is so, why were there large individual differences in the variance task when the item numbers in a set were 2 and 4? Why was the individual difference in the averaging task relatively smaller than it was in the variance task at set sizes of 2 and 4? One possibility is that when the set size is small (e.g., 2 and 4), it is difficult for observers to figure out how variance can be estimated. As a result, observers might estimate variance by taking a stopgap measure, rather than using a particular strategy in performing the task. For example, some observers might use the size difference between items as the indicator for variance; others might use the presence of a seeming outlier. Besides, estimating variance may not be as definitive as estimating the average, particularly when the number of items in a set is small.

It is noteworthy that characteristics of the efficiencies mentioned above are mostly consistent with the results of other visual tasks that involve simultaneously presented stimuli (Barlow, 1978; van Meeteren and Barlow, 1981; Tanaka and Ishiguchi, 2006). This implication is consistent with the previous findings of Tanaka and Ishiguchi (2006). In their study, the sampling strategy is varied across the number of items in a set; the efficiency decreased as the number of lines increased until a range of stable efficiency was reached. The efficiency was stable in 12–20 lines, approximating 50%, while efficiency fluctuated 4–8 lines.

Combining the results of this study and of previous studies, we may infer general sampling characteristics of simultaneous visual tasks such as variance estimation, line discrimination, and texture segmentations, not limited to size averaging. First, human observers may have the visual mechanism required to process the set of multiple items without representing individual properties of each item in a set. Second, when the number of items in a set is 4 and smaller (the number may not be exactly 4 but is presumably around 4), observers inevitably pay focused attention to individual items, whereas when the number of items is larger than 4, whether or not to attend to a particular item in a set becomes an optional strategy for the observer. These predictions are consistent with the subitizing and approximate numerosity literature: when the number is smaller than 3 or 4, observers can correctly provide the exact number of items in a set, while when the set of items is over 4, discrimination accuracy obeys Weber's law.

Future studies are necessary to test the validity of the present results and to reveal the processes behind the extraction of summary statistics in more detail. First, it is necessary to conduct the size averaging task and the variance discrimination task using set sizes larger than 16. Since we predict that the visual summary processing of a large number of items will be similar to texture processing, we need to use the visual array that will be perceived as “texture.” Second, to elaborate the model, it is necessary to examine whether the information of each item in a set is equally weighted or weighted differently, and if so, to specify what may cause different weightings. Third, it is necessary to examine how sampling strategy is determined and whether precision in averaging and variance estimation may improve by learning. There might be individual differences in performance in the way an observer access summary statistics. Fourth, we need to develop an experimental method that can test the ability of visual system to extract summary statistics without using words such as “average,” “variance,” “skewness.” Since usage of these terms presupposes knowledge of the concepts, understanding the task may depend on whether or not observers have the requisite knowledge for adequate concept use. At any rate, a method is needed for developmental and comparative studies.

In summary, this study investigated sampling properties of visual information used by human observers in extracting two types of summary statistics of item sets: average and variance. Our results provide evidence that the extraction of summary statistics may not necessarily require the representation of individual objects with focused attention when the sets of items are larger than 4. The fluctuations of statistical efficiencies across set sizes are consistent with those in other visual tasks such as line discrimination, dot density discrimination, and numerosity discrimination. The extraction of summary statistics of items in a set may share a common mechanism with those visual tasks that involve multiple simultaneous stimuli.

## Author contributions

MT, AI Conceived and designed the experiments. MT, SU Performed the experiments. MT Analyzed the data. AI contributed reagents/materials/analysis tools. AI, MT and SU contributed to the writing of the manuscript.

## Funding

This research was partially supported by a Grant-in-Aid for Scientific Research (C) (40571112) and Scientific Research (B) (15H03462) provided by the Japan Society for the Promotion of Science to the authors.

## Author note

Parts of Experiment 1 and 2 are based on work previously presented at the Cognitive Science Conference and published in the Proceedings of the 36th Annual Conference of the Cognitive Science Society.

### Conflict of Interest Statement

The authors declare that the research was conducted in the absence of any commercial or financial relationships that could be construed as a potential conflict of interest. The reviewer, VN, and handling Editor declared their shared affiliation, and the handling Editor states that the process nevertheless met the standards of a fair and objective review.
