# Supplementary material for: Evidence for a Global Sampling Process in Extraction of Summary Statistics of Item Sizes in a Set
Source: Front Psychol. 2016 May 13;7:711. doi: 10.3389/fpsyg.2016.00711 (PMC4866529; doi:10.3389/fpsyg.2016.00711)
Supplement: Supplementary file 1 [file DataSheet1.pdf]

Appendix: Derivation of each model

It has been proved that a lognormal distribution of circle diameters will produce a Gaussian distribution of discriminable sizes after logarithmic transduction. Thus, when attempting to generate normal distributions of transduced size, we use a lognormal distribution of circle diameters (Solomon, Morgan, & Chubb, 2011).

Discrimination of average size (Task in Experiment 2)

Item sizes in a set are chosen from a lognormal distribution of diameter sizes having mean *D* and SD σ_c. Thus, $Size_{item}$ ($item = 1,2,\ldots,n$) is expressed as follows:

$$Size_{item} \sim \ln N(\ln D, \sigma_c^2) \qquad (1)$$

Global sampling model 1: In GSM1, intrinsic noise, $\sigma_{intrinsic}$, is added to each item in an item set, $Size_{item\_intrinsic}$, and a test item, $Size_{test\_intrinsic}$, independently. Thus, perceived size is expressed as follows:

$$Size_{item\_intrinsic} \sim lnN(\ln Size_{item}, \sigma_{intrinsic}^2)$$

$$Size_{test\_intrinsic} \sim lnN(\ln Size_{test}, \sigma_{intrinsic}^2) \qquad (2)$$

Note that the value of $\sigma_{intrinsic}$ is obtained in Experiment 1.

The average size of the item set, $Size_{ave\_GSM1}$, is expressed as follows:

$$Size_{ave\_GSM1} = \sum_{item}^{n} Size_{item\_intrinsic} / n, \qquad (3)$$

where *n* is the number of items in a set (i.e., set size).

$P\ ((Size_{test\_intrinsic} - Size_{ave\_GSM1}) > 0)$ is obtained and transformed to z_c. Now, d'_I can be calculated by $d'_I = \sqrt{2} z_c$.

Global sampling model 2: In GSM2, sampling noise, $\sigma_{sampleN}$, is added to each item in an item set. Thus, the sampled item size, $Size_{item\_sampleN}$, is expressed as follows:

$$Size_{item\_\_sampleN} \sim \ln N(\ln Size_{item}, \sigma_{sampleN}^2)$$

Note that the value of $\sigma_{sampleN}$ is obtained in Experiment 1.

The average size of the item set, $Size_{ave\_GSM2}$, is expressed as follows:

$$Size_{ave\_GSM2} = \sum_{item}^{n} Size_{item\_sampleN} / n. \qquad (4)$$

$P((Size_{test\_intrinsic} - Size_{ave\_GSM2}) > 0)$ is obtained and transformed to z_c. Now, d'_I can be calculated by $d'_I = \sqrt{2} z_c$.

Limited sampling model (LSM): LSM randomly samples four items from an item set, except for two items sampled in the set size two condition, using the same structures as GSM2. Thus, average size, $Size_{ave\_LSM}$, is expressed as follows:

$$Size_{ave\_LSM} = \sum_{item}^{n} Size_{item\_sampleN} / n,$$

where $n = 2$ *or* 4.

$P(Size_{ave\_LSM} - Size_{test\_intrinsic})$ is obtained and transformed to z_c. Now, d'_I can be calculated by $d'_I = \sqrt{2} z_c$.

Discrimination of variance (Task in Experiment 3)

Item sizes in the standard set and the comparison set are chosen from a lognormal distribution of diameter sizes having mean *D* and SD σ_{stan} for the standard set and σ_{comp} for the comparison set. Thus, $Size_{stan\_item}$ ($item = 1,2,\ldots,n$) and $Size_{comp\_item}$ ($item = 1,2,\ldots,n$) are expressed as follows:

$$Size_{stan\_item} \sim \ln N(\ln D, \sigma_{stan}^2) \text{ and}$$

$Size_{comp\_item} \sim \ln N(\ln D, \sigma_{comp}^2)$, respectively

Global sampling model 1: In GSM1, intrinsic noise, $\sigma_{intrinsic}$, is added to each item in a set. Thus, the perceived size of items in each set is expressed as follows:

$$Size_{stan\_intrinsic} \sim \ln N(\ln Size_{stan}, \sigma_{intrinsic}^2)$$

$$Size_{comp\_intrinsic} \sim \ln N(\ln Size_{comp}, \sigma_{intrinsic}^2)$$

Variance of the standard set, $Size_{stan\_var\_GSM1}$, and that of the comparison set, $Size_{comp\_var\_GSM1}$, is given by the following equations:

$$Size_{stan\_var\_GSM1} = [(Size_{stan1\_intrinsic} - Size_{s\_ave\_GSM1})^2 + (Size_{stan2\_intrinsic} - Size_{s\_ave\_GSM1})^2 + \ldots + (Sizes_{stann\_intrinsic} - Size_{s\_ave\_GSM1})^2]/n,$$

$$Size_{comp\_var\_GSM1} = [(Size_{comp1\_intnois} - Size_{c\_ave\_GSM1})^2 + (Size_{c2\_iintrinsic} - Size_{c\_ave\_GSM1})^2 + \ldots + (Size_{cn\_intrinsic} - Size_{c\_ave\_GSM1})^2]/n,$$

provided $Size_{stan\_ave\_GSM1}$ and $Size_{comp\_ave\_GSM1}$ are given by Equation (3).
$P((Size_{comp\_var\_GSM1} - Size_{stan\_var\_GSM1}) > 0)$ is obtained and transformed to z_c. Now, d'_I can be calculated by $d'_I = \sqrt{2}z_c$.

Global sampling model 2: In GSM2, sampling noise, $\sigma_{sampleN}$, is added to each item in a set. Thus, the sampled size in the standard set, $Size_{stan\_sampleN}$, and the comparison set, $Size_{comp\_sampleN}$, are expressed as follows:

$$Size_{stan\_sampleN} \sim \ln N(\ln Size_{stan}, \sigma_{sampleN}^2) \text{ and}$$

$$Size_{ccomp\_sampleN} \sim \ln N(\ln Size_{comp}, \sigma_{sampleN}^2), \text{ respectively.}$$

Variance of the standard set, $Size_{stan\_var\_GSM2}$, and that of the comparison set, $Size_{comp\_var\_GSM2}$, is given by the following equations:

$$Size_{stan\_var\_GSM2} = [(Size_{stan1\_sampleN} - Size_{stan\_ave\_GSM2})^2 + (Size_{stan2\_sampleN} - Size_{stan\_ave\_GSM2})^2 + \ldots + (Sizes_{stan\_n\_sampleN} - Size_{stan\_ave\_GSM2})^2]/n \text{ and}$$

$$Size_{comp\_var\_GSM2} = [(Size_{comp1\_sampleN} - Size_{comp\_ave\_GSM2})^2 + (Size_{comp2\_sampleN} - Size_{comp\_ave\_GSM2})^2 + \ldots + (Size_{compn\_sampleN} - Size_{comp\_ave\_GSM2})^2]/n,$$

provided $Size_{stan\_ave\_GSM2}$ and $Size_{comp\_ave\_GSM2}$ are given by Equation (4).
$P((Size_{comp\_var\_GSM2} - Size_{stan\_var\_GSM2}) > 0)$ were obtained and transformed to z_c. Now, d'_I can be calculated by $d'_I = \sqrt{2}z_c$.

Limited sampling model (LSM): LSM randomly sampled four items from a set, except for two items sampled in the set size two condition, using the same structures as that of the GSM2 model. Thus, variances of the standard and comparison sets are given by the following equations:

$$Size_{stan\_var\_LSM} = [(Size_{stan1\_sampleN} - Size_{s\_ave\_LSM})^2 + \ldots + (Size_{stan\_n\_sampleN} - Size_{stan\_ave\_LSM})^2]/n \text{ and}$$

$$Size_{comp\_var\_LSM} = [(Size_{comp1\_sampleN} - Size_{comp\_ave\_LSM})^2 + \ldots + (Size_{comp\_n\_sampleN} - Size_{comp\_ave\_LSM})^2]/n,$$

provided $Size_{stan\_ave\_LSM}$ and $Size_{comp\_ave\_LSM}$ are given by Equation (4).
$P((Size_{comp\_var\_GSM2} - Size_{stan\_var\_GSM2}) > 0)$ were obtained and transformed to z_c. Now, d'_I can be calculated by $d'_I = \sqrt{2}z_c$.
